# Detection of Zoonotic Protozoan Enteropathogens in Stray and Owned Dogs with Diarrhea in Trabzon Province Using Microscopic and Molecular Methods

**DOI:** 10.3390/vetsci13101072

**Published:** 2026-10-10

**Authors:** Özlem Koç, Hüseyin Koç, Mustafa Açıcı

**Affiliations:** 1 Department of Aquatic Animal Health, Central Research Institute for Aquaculture, General Directorate of Agricultural Research and Policies, Trabzon 61000, Türkiye; 2 Department of Veterinary Medicine, Vocational School of Maçka, Karadeniz Technical University, Trabzon 61000, Türkiye; 3 Department of Parasitology, Faculty of Veterinary Medicine, Ondokuz Mayıs University, Samsun 55000, Türkiye

**Keywords:** *Cystoisospora*, dog, feces, PCR, RFLP, *Sarcocystis*

## Abstract

Zoonotic protozoan parasites in dogs represent a significant public health concern because of their ability to infect humans. This study aimed to detect and determine the prevalence of zoonotic protozoan infections, including *Balantidium coli*, *Entamoeba histolytica*, *Giardia duodenalis*, *Cryptosporidium* spp., *Sarcocystis* spp. and *Cystoisospora* spp. in stray and owned dogs with diarrhea in Trabzon Province, Türkiye. Fecal samples were obtained from 450 dogs of various ages, sexes, and breeds. Samples were preserved in 70% alcohol for molecular analysis and incubated in 2.5% potassium dichromate for microscopic examination. Parasite DNA was analyzed using polymerase chain reaction (PCR) and identified by restriction fragment length polymorphism (RFLP). Microscopic examination identified the *Cystoisospora ohioensis* group, *Cystoisospora canis*, and *Sarcocystis* spp. in 56 samples (12.44%). Specifically, *Cystoisospora* spp. and *Sarcocystis* spp. were detected in 36 (8.00%) and 20 (4.44%) of the samples, respectively. A mixed infection involving *C. canis* and the *C. ohioensis* group was observed in one sample. Molecular analyses detected *Cystoisospora* spp. DNA in 36 samples (8.00%) and *Sarcocystis* spp. DNA in 20 samples (4.44%). RFLP analysis confirmed that all detected *Cystoisospora* spp. DNA belonged to *C. ohioensis*, while *Sarcocystis* spp. could not be differentiated at the species level. This study reports the first microscopic detection of *Sarcocystis* spp. and *C. ohioensis* in dogs in Trabzon Province and constitutes the first molecular identification of *C. ohioensis* in Türkiye.

## Simple Summary

This study investigated the prevalence of zoonotic parasites in owned and stray dogs with diarrhea in Trabzon. Fecal samples collected from 450 dogs were analyzed using microscopy, PCR, and RFLP methods. Parasites were detected microscopically in 12.44% (56 samples) of the samples; 8.00% (36 samples) of these were identified as *Cystoisospora* spp. and 4.44% as *Sarcocystis* spp. Molecular analyses confirmed that all detected *Cystoisospora* DNA belonged to *Cystoisospora ohioensis*. This study is significant as it marks the first microscopic detection of *Sarcocystis* spp. and *C. ohioensis* in Trabzon and the first molecular-level identification of *C. ohioensis* in Türkiye.

## 1. Introduction

Dogs are domesticated animals that frequently inhabit environments shared with humans. The rising populations of both owned and unowned dogs present potential threats to public health, safety, and the environment [1]. Stray dogs contribute significantly to the transmission of zoonotic diseases by serving as intermediate or definitive hosts for protozoan infections, including Giardiosis, Amoebiosis, Cryptosporidiosis, Cystoisosporosis, Neosporosis, and Hammondiosis. These pathogens pose a public health risk due to their capacity for transmission to other animals and humans [2,3].

*Giardia* is a globally prevalent parasite that infects humans, cattle, domestic and wild animals, and aquatic species [4]. A meta-analysis of 127 studies on giardiosis in dogs revealed an overall prevalence rate of 15.2% [5]. *Giardia* species have several genotypes, particularly strains A and B, which are zoonotic and can infect both people and animals. The frequency of strain A in canines renders them a possible vector for infection, hence amplifying the significance of giardiosis in public health considerations [6,7].

The prevalence of strain A in dogs makes them potential carriers of infection, thereby increasing the public health significance of giardiosis. Entamoebosis is an infection characterized by moderate to severe gastroenteritis caused by *Entamoeba histolytica*, a free-living, facultative parasite [8]. Approximately 50 million individuals globally contract the parasite annually, resulting in over 100,000 fatalities [9]. Researchers have primarily studied *E. histolytica* prevalence in humans in Türkiye, with reported rates ranging from 0.4% to 18.4% [10]. A study in Van revealed a 7.24% prevalence of *Entamoeba* spp. in the feces of 152 stray dogs using microscopic analysis [11].

Administering the right medication to sick dogs is essential to prevent the spread of this zoonotic parasite [12]. Cryptosporidiosis is a worldwide zoonotic infection caused by species of *Cryptosporidium* that affects the digestive and respiratory systems of birds, mammals, fish, and reptiles [13]. Dogs are most commonly infected with *C. canis*, although occasional infections with *C. parvum*, *C. meleagridis*, *C. ubiquitum*, and *C. andersoni* have also been reported. The reported prevalence of *Cryptosporidium* spp. in canines ranges from 0% to 44.8% [14]. In Van, a study identified *Cryptosporidium* spp. in 15.78% of fecal samples from 152 stray dogs using the carbol fuchsin staining method [11].

*Cystoisospora* infections, formerly known as *Isospora*, are coccidial diseases commonly observed in juvenile canines. These infections manifest as intestinal disorders, including colitis and enteritis, and may result in growth retardation, weight loss, and diarrhea. Puppies with compromised immune systems are particularly vulnerable to these conditions. *Cystoisospora* represents a prevalent form of coccidiosis that contributes to developmental abnormalities in young dogs [15]. In Türkiye, research on zoonotic gastrointestinal protozoan infections in dogs has largely focused on microscopic examination, with limited molecular studies. Given the importance of these protozoan pathogens to both animal and public health, comprehensive investigations into their prevalence in Türkiye are warranted.

This study was carried out in the Trabzon region and aimed to investigate the presence of gastrointestinal protozoa in dogs at both microscopic and molecular levels, evaluate the prevalence of zoonotic and host-specific veterinary protozoan infections, and contribute to the development of targeted protective and preventive strategies for animal and public health.

## 2. Materials and Methods

Study materials: We conducted a cross-sectional study to evaluate fecal samples from 450 dogs presenting with diarrhea in Trabzon province. Convenience sampling was employed. We recruited owned dogs during visits to local veterinary clinics, whereas we sampled stray dogs from animal shelters and urban areas throughout the province. The primary inclusion criterion was clinical diarrhea at the time of sampling; dogs without diarrhea were excluded. We stratified by housing status, breed, sex, and age (0–6 months, 6–12 months, and over 12 months). Of the 450 dogs sampled, 155 were owned, and 295 were strays. Table 1 provides a detailed demographic distribution of the sampled dogs.

Examination of fecal samples: We collected two fecal samples from each dog in sterile plastic containers with screw caps for microscopic and molecular analysis, and assigned each sample a unique protocol number. A detailed data record table was created to include the age, breed, sex, and housing conditions of the dogs. We conducted microscopic analysis using 2.5% potassium dichromate in fecal samples, while we treated samples for molecular analysis with 70% alcohol. All samples were transported to the laboratory under cold chain; molecular analysis samples were stored at −20 °C, while microscopic examinations were performed on the same day.

The Nativ–Lugol method was used for direct microscopic analysis of stool samples. In the Nativ method, stool samples were homogenized with isotonic saline, placed on a slide, covered with a coverslip, and trophozoites and cysts of *Giardia duodenalis*, *E. histolytica* and *Balantidium coli* were examined under a light microscope at 10×–40× magnification [5,8]. *Cystoisospora* spp., *Toxoplasma*, *Neospora*, *Hammondia* oocysts, and *Sarcocystis* sporocysts were evaluated with a sugar solution with a density of 1.27; other protozoa were evaluated using the 33% zinc sulfate (ZnSO_4_) flotation method [16].

Kinyoun’s modified acid-fast staining method was used to identify *Cryptosporidium* spp. oocysts and slides were examined at 100× magnification using immersion oil [17]. To confirm the microscopic findings and achieve species-level identification, molecular analyses were exclusively performed on the 56 samples that tested positive during the initial microscopic screening. For these microscopically positive samples, DNA isolation was performed using the QIAamp Fast DNA Stool Mini Kit (Qiagen^®^, Hilden, Germany) [18]. Before isolation, the samples were exposed to liquid nitrogen and boiling water for 1 min each, and this process was repeated eight times to break down the cell walls of cysts and oocysts; all procedures were carried out in a biosafety cabinet [19]. The PCR-RFLP method was applied for molecular identification [20]. For *Cystoisospora* spp., a 450 bp target region was amplified using the ITS-1 gene region with ITSGF and ITSR2 primers [21]. The PCR mixture was prepared as detailed in Table 2, and amplification was performed using a thermal cycler (Applied Biosystems, SimpliAmp, Waltham, MA, USA). Amplification products were analyzed by electrophoresis in a 1.5% agarose gel. Two bands of 327 bp and 116 bp, specific to *C. ohioensis*, were obtained following digestion with the AluI restriction enzyme. The 18S rRNA region for *Sarcocystis* spp. was amplified by semi-nested PCR [22], and species differentiation was achieved by analyzing the fragments generated by DraI, MboII, RsaI, and SspI enzymes using gel electrophoresis [23].

Due to resource and infrastructural limitations during the study period, formal positive controls (reference genomic DNA) and negative controls were not included in the PCR amplification and RFLP procedures. Consequently, molecular results were interpreted strictly in conjunction with the initial microscopic findings.

**Statistical assessment:** Statistical analyses were conducted using IBM SPSS Statistics 20.0 software. The chi-square test evaluated differences among groups based on housing status, breed, age, and gender. Prevalence rates were calculated with a 95% confidence interval (CI), and odds ratios (OR) were determined. Statistical significance was defined as *p* < 0.05 and *p* < 0.01.

## 3. Results

**Microscopic examination results of fecal samples:** Among the 450 analyzed canine fecal samples, 12.44% (56 out of 450) tested positive for oocysts and sporocysts. *Cystoisospora* spp. oocysts were identified in 8.00% (36 out of 450) (95% CI: 5.60–10.80) of the positive samples (Table 3).

**Figure 1:**
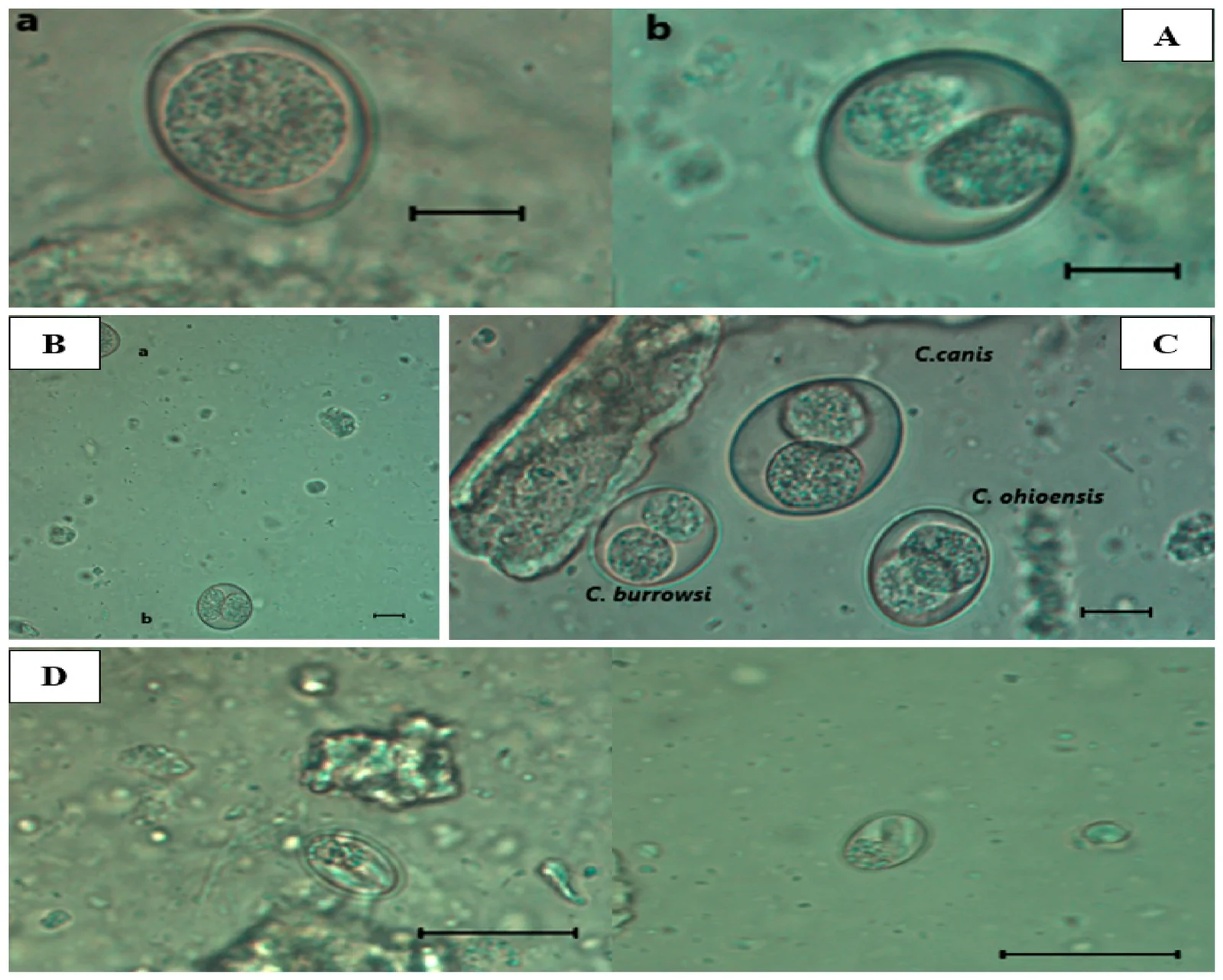
Detection of protozoan oocysts and sporocysts in canine feces: (**A**) *Cystoisospora ohioensis* oocyst, unsporulated (a) and sporulated (b) oocysts (**B**) *C. burrowsi* oocyst, (**C**) *C. canis* oocyst, (**D**) *Sarcocystis* spp. sporocyst (Scale bar: 10 µm for (**A**–**C**); 10 µm for (**D**); Magnification: ×40).

Microscopic analysis detected oocysts of *Cystoisospora canis* and the *Cystoisospora ohioensis* group in 36 stool samples. Morphological measurements of *C. ohioensis* group oocysts ranged from 17 to 25 × 16 to 20 μm (Figure 1A,B). The *C. ohioensis* group exhibited the highest prevalence among identified species, and one fecal sample demonstrated a mixed infection of *C. canis* with the *C. ohioensis* group (Figure 1C). Oocysts of *C. canis* measured 32 × 29 μm. Statistical analysis indicated a significant difference in prevalence between *C. canis* and the *C. ohioensis* group (*p* < 0.05). Microscopic examination also identified *Sarcocystis* spp. sporocysts in 20 fecal samples. At 40× magnification, the ellipsoidal sporocysts measured 18–10.5 × 10–8 μm (average 15 × 10 μm) (Figure 1D).

Microscopic examination using the centrifuge flotation method with zinc sulfate solution did not detect *Giardia* spp. trophozoites or cysts. Additionally, *Cryptosporidium* spp. oocysts were not observed in samples analyzed with Kinyoun’s Modified Acid-Fast Staining technique. Similarly, trophozoites and cysts of *Entamoeba histolytica* and *Balantidium coli* were absent in the fecal samples analyzed.

**Figure 2:**
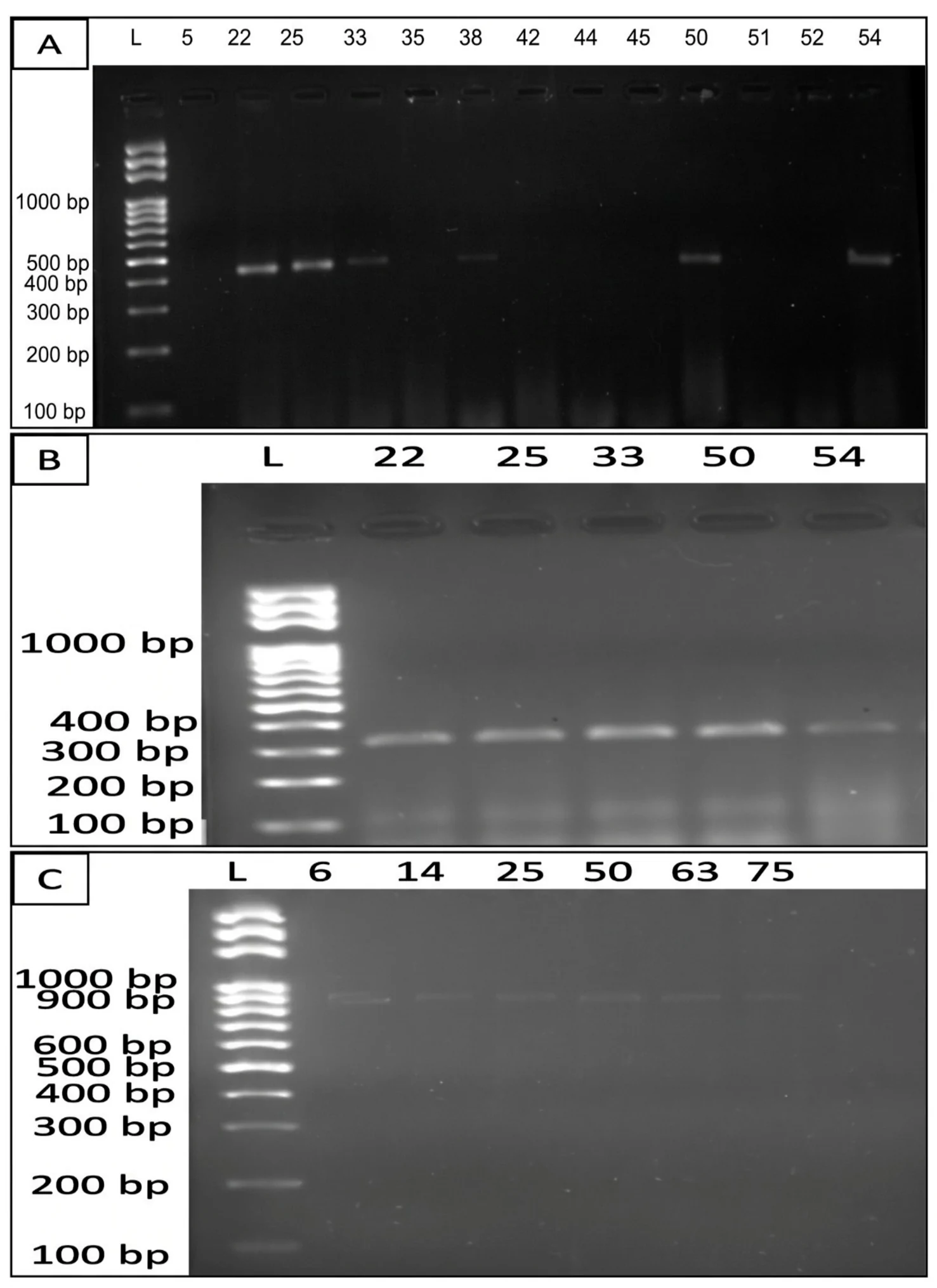
Band images derived from PCR and PCR-RFLP analyses: (**A**) PCR bands resulting from the amplification of 450 bp of the ribosomal DNA ITS-1 gene region of *Cystoisospora* spp. (L: Ladder, samples 5–54). (**B**) Gel image of PCR-RFLP analysis of the rDNA ITS1 region for the differentiation of *Cystoisospora* species (L: 200 bp ladder, 22–54 *C. ohioensis* isolates). (**C**) PCR amplification yielded bands of 900 bp corresponding to the 18S rRNA gene region, confirming the presence of *Sarcocystis* spp. (L: 200 bp ladder, samples 6–75).

**Results of molecular analysis:** Detection of *Cystoisospora ohioensis* was achieved by amplifying approximately 450 base pairs of a target region using PCR with primers specific to the ITS1 gene region of ribosomal *Cystoisospora* spp. DNA. Amplification was successful in all 36 samples previously identified as positive for *Cystoisospora* oocysts by microscopy (Figure 2A). PCR-RFLP analysis with the AluI restriction enzyme was used to differentiate species in PCR-positive samples, identifying 36 samples (8.00%) (95% CI: 5.60–10.80) as *C. ohioensis* based on the resulting band profile. The AluI enzyme cleaved *C. ohioensis* DNA into two fragments of 326 and 116 base pairs (Figure 2B). Initial semi-nested PCR assays for *Sarcocystis* spp., using the 18S2L/18S2H primer pair targeting the 18S rRNA gene region, did not yield amplification in any of the 20 stool samples, resulting in negative PCR outcomes. Subsequently, a secondary PCR analysis with the 18S9L/18S1H primer pair, which amplifies 900 base pairs of the relevant gene region, detected weak bands of *Sarcocystis* spp. DNA in all 20 samples that were microscopically positive for *Sarcocystis* sporocysts (Figure 2C).

PCR-RFLP analysis using DraI, MboI, RsaI, and SspI restriction enzymes was performed to identify *Sarcocystis* spp. at the species level. However, the cleavage process did not yield a discernible RFLP band profile, which prevented species-level identification. Consequently, *Sarcocystis* spp. identification was restricted to the genus level.

**Examination of risk determinants for *Cystoisospora ohioensis* infection:** Risk factors for *Cystoisospora ohioensis* infection in canines were evaluated across multiple characteristics. Infection prevalence varied significantly among age groups (*p* < 0.05), with the highest rates observed in young dogs and a decline noted with increasing age. This pattern suggests a strong association between age and infection. No significant differences were found between male and female dogs, indicating that gender does not influence transmission. Similarly, infection rates did not differ significantly between owned and unowned dogs. A statistically significant difference was observed among breeds (*p* < 0.05), with the highest infection rate reported in the Labrador breed (Table 4). However, the small sample size in certain breed categories (for example, Labrador, n = 4), resulting in wide 95% confidence intervals, and the inability to control for confounding variables such as age or housing status, limit the reliability of breed-specific comparisons. Consequently, these results do not support breed as an independent risk factor for transmission.

**Examination of risk factors associated with *Sarcocystis* infection:** The prevalence of *Sarcocystis* spp. infection in definitive host dogs was 4.44% (95% CI: 2.70–6.70). The highest infection rate occurred in the 6–12 months age group, while lower rates were observed in dogs aged 12 months and older. Infection was rare in dogs aged 0 to 6 months. The difference in infection rates among age groups was statistically significant (*p* < 0.05). Although male dogs exhibited a higher infection rate than female dogs, the effect of gender on prevalence was not statistically significant. Unowned dogs had a higher prevalence of infection compared to owned dogs, but this difference was also not statistically significant. Analysis by breed indicated that Labrador Retrievers had the highest infection rate, followed by Anatolian Shepherd Dogs and German Shepherd Dogs; no infections were detected in the Tonya Poodle breed. While univariate statistical analysis revealed a significant difference among breed groups (*p* < 0.05) (Table 5), this finding should be interpreted with caution. The uneven distribution and small sample sizes within breed categories produced wide confidence intervals, limiting the ability to draw definitive epidemiological conclusions.

## 4. Discussion

Protozoan parasites infecting the digestive tracts of dogs pose significant risks to animal health, with several species also constituting notable public health threats due to their zoonotic potential [24]. Epidemiological differentiation between true zoonotic agents and strictly host-specific veterinary pathogens remains essential. Analysis of 450 dog fecal samples revealed oocysts and sporocysts from four distinct protozoan species in 12.44% (n = 56) of the samples. Based on molecular analyses, the identified species included *Cystoisospora ohioensis* and *Sarcocystis* spp.

Research on the prevalence of *Entamoeba histolytica* in dogs reports rates of 36.6% in Malaysia [8], 4.8% in Brazil [25], between 15.4% and 37.1% in Nigeria [26,27], and 15.6% in Pakistan [28]. Additionally, a study of stray dogs in Van province, Türkiye, found a detection rate of 11.48% for *Entamoeba* spp. [11]. *Entamoeba histolytica* was not detected in any of the samples analyzed in this study. This absence may be attributed to specific environmental and epidemiological factors.

Reported prevalence rates of *Giardia* spp. vary widely, ranging from 2.6% to 57.7% in Brazil [3,29], 9.67% in Iraq [30], 5.3% in China [31], 41% in Italy [6], and 9.9% in Nepal [2]. In Germany, *Giardia intestinalis* was detected at a rate of 29%, with Assemblage C and D isolates identified by sequence analysis [32]. In Türkiye, *Giardia* spp. prevalence rates have been reported as 2.48% in Konya [33], 2–22% in Van [34], and 35.2% in Kayseri using the ELISA method [35]. No *Giardia* spp. cysts were detected in the present study. Environmental factors, canine living conditions, and variations in diagnostic methods may have influenced these findings.

Reported prevalence rates of *Cryptosporidium* spp. include 26.2% in Brazil [25], 12.9% in Iraq [30], 3.8% in Iran [36], and 1.16–5.51% in Korea [37]. In Türkiye, *Cryptosporidium* spp. prevalence was reported as 26.23% in Van [11] and 3% in Diyarbakır [38]. The present study did not detect *Cryptosporidium* spp.

Studies on *Cystoisospora* spp. report prevalence rates of 0.2% in Finland [39], 2.8% in Germany [40], 4.39% in Portugal [41], and 5.1% in Great Britain [42]. In Türkiye, prevalence ranges from 1% to 8.55% in Van [11,34] and reaches 26.4% in Konya [33]. The present study provides the first molecular-level epidemiological data on *Cystoisospora* spp. in this context, with a prevalence rate of 8.00% (95% CI: 5.60–10.80). Morphological differentiation of *Cystoisospora* species remains challenging due to overlapping oocyst dimensions, particularly between *C. ohioensis* and *C. burrowsi*, which are collectively referred to as the *C. ohioensis* group during microscopic evaluation. The PCR-RFLP assay targeting the ITS-1 gene region classified these isolates as *C. ohioensis*. However, the taxonomic limitations of RFLP should be acknowledged; although it offers greater resolution than microscopy, definitive differentiation of closely related cryptic species within the *C. ohioensis* complex may require advanced DNA sequencing techniques.

Previous studies have reported *Sarcocystis* spp. prevalence rates of 0.03% in Germany [40], 7.2% in Nepal [2], 8% in Ankara, Türkiye [43], and 19.96% in Van [11]. In the present study, the prevalence of *Sarcocystis* spp. was determined to be 4.44% (95% CI: 2.70–6.70) using molecular methods. A primary limitation of the molecular analysis was the inability of the RFLP assay to differentiate *Sarcocystis* at the species level with the selected restriction enzymes (DraI, MboI, RsaI, and SspI). This limitation likely resulted from weak initial PCR amplification, indicating a low concentration of parasitic DNA in the samples, as well as a possible lack of sufficient polymorphic sites within the amplified 18S rRNA fragment for these enzymes. Therefore, identification was restricted to the genus level (*Sarcocystis* spp.). As dogs are definitive hosts for multiple *Sarcocystis* species, each with distinct life cycles and varying public health significance, the specific zoonotic potential of the isolates detected in this study cannot be determined without species-level identification. Additionally, the study was constrained by the omission of standard positive and negative controls during PCR due to resource limitations. Nevertheless, molecular analyses were used exclusively as a confirmatory tool on samples previously diagnosed as positive by microscopy, which minimized the risk of interpreting potential cross-contamination as false-positive molecular results.

The prevalence of protozoan enteropathogens in dogs in Trabzon province was assessed. Prevalence rates for some species aligned with previous studies, while others were detected at lower levels. The application of molecular methods enabled the identification of species that are challenging to distinguish using morphological characteristics alone.

A limitation of the present study concerns the epidemiological risk factor analysis. The sampling strategy focused exclusively on dogs with clinical diarrhea, resulting in a cohort that does not represent the general canine population in the region. Therefore, the identified risk determinants, such as age, breed, and housing status, reflect statistical associations with protozoan infections only within this symptomatic subpopulation. These findings require cautious interpretation, as they may not accurately represent risk determinants for the broader, asymptomatic canine population in Trabzon province.

Another methodological limitation of this study is the exclusive use of microscopy as the primary screening method. Molecular analyses were performed only on samples that tested positive by microscopy for confirmation, which may have resulted in false-negative findings in cases with low oocyst or sporocyst shedding. Given that microscopy typically exhibits lower analytical sensitivity than PCR, the actual prevalence of protozoan infections in the studied population may be underestimated.

Furthermore, the statistical associations identified in this study, especially those concerning dog breeds, are derived from univariate analyses and may be influenced by confounding variables. The uneven distribution of the sampled population and the limited sample sizes within certain breed categories substantially reduce the statistical power of these comparisons, as indicated by the wide confidence intervals for the calculated Odds Ratios. Therefore, the observed breed differences should not be regarded as definitive evidence of genetic predisposition without confirmation from larger, multivariable epidemiological studies.

## 5. Conclusions

This study investigated the prevalence of key zoonotic and veterinary gastrointestinal protozoa in diarrheic dogs in Trabzon province. Only dog-specific veterinary pathogens, including *Cystoisospora ohioensis*, *C. canis*, and *Sarcocystis* spp., were identified. These findings provide the first molecularly confirmed epidemiological data for *C. ohioensis* and *Sarcocystis* spp. infections in dogs in Türkiye. Interpretation of these results should consider certain diagnostic limitations. Reliance on microscopy as the sole primary screening method may have underestimated the true prevalence due to potential false negatives in subclinical infections. Additionally, while molecular characterization via PCR-RFLP successfully identified *C. ohioensis*, it did not achieve species-level identification for *Sarcocystis*, preventing definitive conclusions regarding the specific epidemiological significance or zoonotic potential of these isolates. Despite these limitations, the results underscore the need for continued veterinary surveillance, improved environmental hygiene, and prevention of raw meat consumption by dogs to disrupt parasite life cycles and protect animal health. The molecular presence of *C. ohioensis* in dogs in Türkiye is confirmed for the first time. Although potentially zoonotic agents targeted in this study were not detected, the identification of *Cystoisospora* and *Sarcocystis* highlights the ongoing need for veterinary preventive measures. Key strategies include improving routine care of dogs, enhancing environmental hygiene to reduce *Cystoisospora* transmission, and preventing dogs from consuming raw meat to disrupt the predator–prey life cycles of *Sarcocystis* species.

## Figures and Tables

**Table 1:** Number of feces examined by dog breed and age groups.

Dog Breed	Age Group	Number of Dogs Examined
Hybrids	0–6 months	203
	6–12 months	83
	>12	34
Anatolian Shepherd	0–6 months	45
	6–12 months	7
	>12	11
German Shepherd	0–6 months	36
	6–12 months	2
	>12	9
Tonya Poodle	0–6 months	8
	6–12 months	2
	>12	6
Labrador	0–6 months	2
	6–12 months	1
	>12	1
Total		450

**Table 2:** PCR amplification protocols.

Protocols	Cycle No	Denaturation	Attachment	Extension
For *Cystoisospora* spp.	Beginning	2 min, 94 °C	-	-
	40 cycles	45 s, 94 °C	45 s, 55 °C	45 s, 72 °C
	Last cycle	-	-	10 min, 72 °C
	The end	-	4 °C	-
For *Sarcocystis* spp.	Beginning	3 min, 94 °C	-	-
	35 cycles	30 s, 94 °C	30 s, 60 °C	30 s, 72 °C
	Last cycle	-	-	10 min, 72 °C
	The end	-	4 °C	-

**Table 3:** Morphologically detected *Cystoisospora* oocysts in dog feces and their prevalence.

Species	Number of Mixed Infected Feces	Prevalence	95% CI for Prevalence (%)
*C. ohioensis* group (*C. ohioensis* + *C. burrowsi*)	35	36/450 (7.78%)	5.50–10.70
*C. ohioensis* group + *C. canis*	1	1/450 (0.22%)	0.00–1.20
Total	36	36/450 (8.00%)	5.60–10.80

CI: Confidence Interval.

**Table 4:** Analysis of *Cystoisospora ohioensis* infection by age, gender, breed and feeding style.

Variable	n	Positive	Prevalence (%)	95% CI for Prev. (%)	Odds Ratio (OR)	Chi-Square
Age						
0–6 months	294	29	9.86	6.70–13.80	Ref.	*X*^2^ = 4.402; *p* < 0.05
6–12 months	95	5	5.26	1.70–11.80	0.50	
>12	61	2	3.27	0.40–11.30	0.30	
Gender						
Male	213	20	9.38	3.90–10.70	Ref.	*X*^2^ = 1.061; *p* > 0.05
Female	237	16	6.75	5.80–14.10	1.43	
Lifestyle						
Owned Dog	155	12	7.74	4.00–13.10	Ref.	*X*^2^ = 0.021; *p* > 0.05
Stray Dog	295	24	8.13	5.30–11.80	1.05	
Breed						
Hybrid	320	15	4.68	2.60–7.60	Ref.	*X*^2^ = 17.284; *p* < 0.05
Anatolian Shepherd	63	10	15.87	7.90–27.30	3.83	
German Shepherd	47	8	17.02	7.60–30.80	4.17	
Tonya Poodle	16	2	12.50	1.50–38.30	2.90	
Labrador	4	1	25.00	0.60–80.50	6.77	
Total	450	36	8.00	5.60–10.80		

n: number of animals; CI: Confidence Interval; Ref.: Reference category for OR calculation.

**Table 5:** Analysis of *Sarcocyctis* spp. infection by age, gender, breed and feeding style.

Variable	n	Positive	Prevalence (%)	95% CI for Prev. (%)	Odds Ratio (OR)	Chi-Square
Age						
0–6 months	294	2	0.68	0.10–2.40	Ref	*X*^2^ = 30.928; *p* < 0.05
6–12 months	95	13	13.68	7.40–22.20	23.14	
>12	61	5	8.19	2.70–18.10	13.03	
Gender						
Male	213	11	5.16	1.70–7.10	Ref	*X*^2^ = 0.493; *p* > 0.05
Female	237	9	3.79	2.60–9.00	1.37	
Lifestyle						
Owned Dog	155	3	1.93	0.40–5.50	Ref	*X*^2^ = 3.505; *p* > 0.05
Stray Dog	295	17	5.76	3.40–9.00	3.09	
Breed						
Hybrid	320	10	3.12	1.50–5.60	Ref	*X*^2^ = 10.279; *p* < 0.05
Anatolian Shepherd	63	6	9.52	3.50–19.60	3.26	
Germen Shepherd	47	3	6.38	1.30–17.50	2.11	
Tonya Poodle	16	0	0.00	0.00–20.60	NA	
Labrador	4	1	25.00	0.60–80.50	10.33	
Total	450	20	4.44	2.70–6.70		

n: number of animals; CI: Confidence Interval; Ref: Reference category for OR calculation; NA: Not Applicable.

## Data Availability

The data supporting the findings of this study are available from the corresponding author upon reasonable request.
